# SeXY chromosomes and the immune system: reflections after a comparative study

**DOI:** 10.1186/s13293-019-0278-y

**Published:** 2020-01-14

**Authors:** Irene Meester, Edgar Manilla-Muñoz, Rafael B. R. León-Cachón, Gustavo A. Paniagua-Frausto, Diego Carrión-Alvarez, C. Orelli Ruiz-Rodríguez, Ximena Rodríguez-Rangel, Joyce M. García-Martínez

**Affiliations:** Ciencias Básicas, Escuela de Medicina, Universidad de Monterrey, Av. Ignacio Morones Prieto 4500 Pte., 66238 San Pedro Garza García, Nuevo León México

**Keywords:** Genetics, Immunity, Sex difference, X chromosome, Y chromosome, DDX3Y, KDM5D, MSL3, UTY

## Abstract

**Background:**

Sex bias in immune function has been contributed in part to a preponderance of immune system-related genes (ISRG) on the X-chromosome. We verified whether ISRG are more abundant on the X chromosome as compared to autosomal chromosomes and reflected on the impact of our findings.

**Methods:**

Consulting freely accessible databases, we performed a comparative study consisting of three complementary strategies. First, among coding X/Y-linked genes, the abundance of ISRG was compared to the abundance of genes dedicated to other systems. Genes were assigned considering three criteria: disease, tissue expression, and function (DEF approach). In addition, we carried out two genome-wide approaches to compare the contribution of sex and autosomal chromosomes to immune genes defined by an elevated expression in lymphatic tissues (LTEEG approach) or annotation to an immune system process, GO:0002376 (GO approach).

**Results:**

The X chromosome had less immune genes than the median of the autosomal chromosomes. Among X-linked genes, ISRG ranked fourth after the reproductive and nervous systems and genes dedicated to development, proliferation and apoptosis. On the Y chromosome, ISRG ranked second, and at the pseudoautosomal region (PAR) first. According to studies on the expression of X-linked genes in a variety of (mostly non-lymphatic) tissues, almost two-thirds of ISRG are expressed without sex bias, and the remaining ISRG presented female and male bias with similar frequency. Various epigenetic controllers, X-linked *MSL3* and Y-linked *KDM5D* and *UTY,* were preferentially expressed in leukocytes and deserve further attention for a possible role in sex biased expression or its neutralisation.

**Conclusions:**

The X chromosome is not enriched for ISRG, though particular X-linked genes may be responsible for sex differences in certain immune responses. So far, there is insufficient information on sex-biased expression of X/Y-linked ISRG in leukocytes to draw general conclusions on the impact of X/Y-linked ISRG in immune function. More research on the regulation of the expression X-linked genes is required with attention to 1) female *and* male mechanisms that may either augment or diminish sex biased expression and 2) tissue-specific expression studies.

## Background

Men and women differ in their susceptibility to infectious diseases [1–4], response to vaccines [5], and autoimmune diseases [6, 7]. Though behavioural differences partly explain sex bias in infection susceptibility [8], sex differences in the immune response in animal models under controlled laboratory conditions indicate the role of biological differences [9]. Thus, a sex bias in the immune system seems at least as important. In general, females are more immunocompetent and have a higher leukocyte count than males [10]. Furthermore, type 1 helper T cells (Th1) and the cellular immune response predominate in men, whereas the Th2-controlled antibody-mediated immune response predominates in women [7, 11]. Sex hormones may have a role in regulating the immune response [7, 12–14], but hormonal intervention treatment in the clinic does not always yield the results observed in preclinical animal studies. Furthermore, a sex bias in susceptibility to certain autoimmune disease is observed in pre-puberty children [6], which suggests that other factors play a role. A logical alternative explanation are the sex chromosomes. In 2008, a list of 79 X-linked genes with a possible role in sex-based differences in immune responses was presented [15]. Though the selection criteria for genes to be on the list were not mentioned, the list of X-linked immune genes was well-received by the scientific community and reinforced by studies that associated X-linked immune genes with autoimmune diseases and immune responses [9, 16]. With time, the interpretation of this list changed from the suggestion that X-linked immune genes may have a role in sex differences in the immune response to the interpretation that the *number* of X-linked immune genes may explain sex differences in the immune response to the perception that the X chromosome contains “the largest number of immune-related genes of the whole human genome” [17]. However, as far as we know, the X chromosome has never been compared with autosomal chromosomes with respect to the absolute or relative amount of ISRG. Likewise, the absolute or relative number of X-linked genes dedicated to the immune system or other systems have not been compared. As far as we know, this is the first comparative study to verify whether ISRG are enriched on the X chromosome. Next, we reflect on the functional impact of our findings.

## Methods

### Study design

**Fig. 1:**
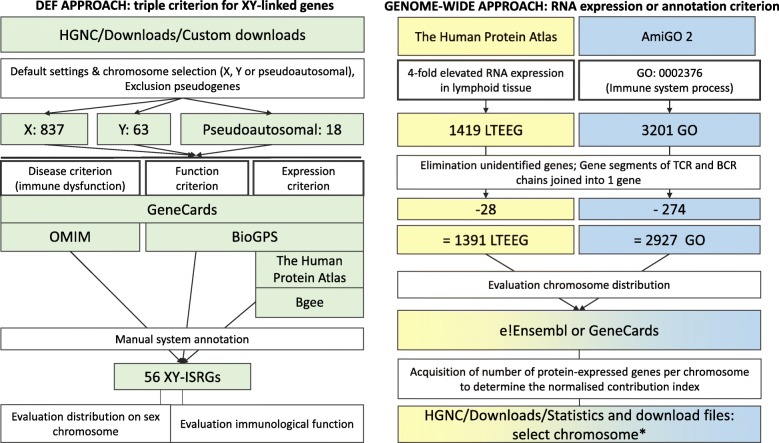
Three comparative approaches to evaluate the relative abundance of human X/Y-linked coding immune genes. DEF approach: Genes on X and Y chromosomes were annotated based on three criteria: disease, tissue expression, and function (DEF approach). Details are explained in the Methods section. Genome-wide genes were selected by either **a**) an elevated expression in lymphoid tissue (LTEEG approach) or **b**) the ´Immune System Process´-annotation, GO:0002376 (GO approach), followed by an analysis of the distribution of the LTEEG and GO genes over the chromosomes. Data were obtained from the following databases: HGNC [18], e!Ensembl [19], GeneCards [20] (Genomics, Function, Expression, and Disorders), BioGPS (U133A GeneAtlas, Primary Cell Atlas) [21], the Human Protein Atlas [22], Bgee [23], and AmiGO 2 [24]

For this comparative study we applied three approaches to verify the relative abundance of protein-expressed sex chromosome-linked genes (X/Y-genes) that seemed especially dedicated to the immune system (Fig. 1). The first approach was limited to X/Y-genes and compared the number of X/Y-genes dedicated to the immune system with the number of X/Y-genes dedicated to other systems. System annotation was done manually based upon convincing compliance with at least one of the following criteria: 1) disease association, 2) preferential tissue expression, and 3) a system-specific function (DEF approach). The disease criterion for the ISRG annotation considered susceptibility to infections, allergies, autoimmune diseases, and immune deficiencies, but susceptibility to cancer was not considered because of the interference from oncogenes and proto-oncogenes. X-linked genes dedicated to other systems provided an endogeneous comparative context. The second approach considered genome-wide genes with a four-fold elevated expression in lymphoid tissues (LTEEG) and compared the number of LTEEG on sex chromosomes with the number of LTEEG on autosomal chromosomes. The third approach only differed from the second for the criterion, which in this case was the annotation to an “immune system process” (GO:0002376).

### Determination of the relative abundance of X/Y-linked ISRG

Full lists of protein-expressed genes on the X chromosome, Y chromosome, and pseudoautosomal regions (PAR) were downloaded from the HUGO Gene Nomenclature Committee (HGNC) database [18] in May 2018 using the ´Custom download option´, with the default option set plus ´Name Synonyms´ in combination with the selected chromosome X, Y, or PAR. Exclusion criteria for downloaded genes on the X and Y chromosome were the identification as pseudogenes and non-coding RNA genes. Furthermore, PAR-listed genes without a pseudoautosomal character, *i.e*. not present on both sex chromosomes, were not considered to be PAR genes (Additional file 1).

From May 2018-May 2019, we collected information on the three DEF criteria for each X/Y-linked gene from a variety of freely accessible databases [20–22, 25, 26] as specified in Fig. 1. Two immunologists independently evaluated the information on each X-linked and Y-linked gene. A pre-selection of ISRG that still included doubtful cases (Additional file 2) passed through the ´Bgee filter´ to confirm or discard ISRG annotation. Doubtful cases seemed to have some importance for the immune system, but did not convincingly comply with any DEF criterion. Especially the expression data from the various databases tended to be inconsistent. The Bgee database [23] presents tissue expression data as a list of tissues that are ordered according to expression level. The criterion for Bgee data was that three lymphoid tissues should be ranked among the first ten, and at least two among the first five. Otherwise, the gene was apparently more abundantly expressed in non-lymphoid tissues. When a gene could not be assigned convincingly to a system, the gene was assigned to ´Basal/ubiquitous/unknown´. The final decision was reached in common agreement by the two immunologists.

To determine whether the X chromosome should be considered enriched for ISRG, an internal reference strategy was used by comparing the absolute number and proportion of ISRG with those of X-linked coding (*i.e.* protein-expressed) genes dedicated to other body systems, followed by a ranking mechanism. As the category ´Basal/ubiquitous/unknown´ was used for any gene that could not be convincingly assigned to a specific system, this category did not participate in the ranking. This relative abundance analysis was also applied to Y-linked and PAR-linked genes. Furthermore, for Y- and PAR-linked genes, a retention ratio was determined. The retention ratio is the ratio of the number of Y-linked or PAR-linked ISRG as a fraction of the number of X-linked ISRG, *i.e.* nY/nX and nPAR/nX, respectively.

### Immune function analysis of ISRG

The X/Y-linked ISRG were grouped according to function with the following options: 1) proliferation/apoptosis, 2) B cell function, 3) T cell function, 4) leukocyte distribution, 5) innate immune system, 6) immune regulation, 7) signal transduction, 8) antigen presentation, 9) tolerance, and 0) unknown function. Genes that resulted as single members of a group were regrouped, most often to immune regulation. A Venn diagram was created with the free tool Metachart [27] and manually corrected.

The gene locus was used to determine the distribution of ISRG over the sex chromosomes.

For each ISRG, data on X chromosome inactivation (XCI), XCI escape and/or sex-biased expression in a variety of tissues and cell lines were obtained from the supplemental data of the studies of Balaton *et al*. and Tukiainen *et al.* [28, 29]. In the study by Tukiainen *et al.,* sex-biased expression data on ISRG were evaluated for 681 genes from 29 tissue types or cell types from 449 persons [29], *i.e.* these expression data are not specific for lymphoid tissue.

### Determination of the relative abundance of X/Y-linked LTEEG

We obtained LTEEG via the ´Tissue atlas´ option from ´The Human Protein Atlas´ database [22, 26] on October 28, 2019 by selecting the option ´Lymphoid tissue´ organ (*i.e.* thymus, spleen, tonsil, lymph node, and appendix) and the *number* of ´elevated genes´ (*i.e.* n = 1419) (Fig. 1). The Human Protein Atlas annotates a gene as elevated when a particular tissue or organ expresses mRNA at least four-fold higher as compared to other tissues. Gene names that were not recognized by other databases (so that their locus could not be obtained) were eliminated (n = 28). Of the resulting 1391 LTEEG genes, the loci were obtained from e!Ensembl [19], using the option BioMart, or GeneCards [20]. The loci were used to determine the distribution of LTEEG over the chromosomes. For each chromosome, the relative contribution to the total LTEEG number was calculated as a percentage. To correct for the variety in chromosome size and gene density, the proportion of LTEEG among coding genes (*i.e.* protein-encoding genes) was determined for each chromosome. Hereto, first the distribution of coding genes over the chromosomes was obtained from the HGNC database with the option ´Statistics and download files´ and the selection a specific chromosome. As this option was not available for the PAR region, we used the number of protein-expressed genes downloaded for the DEF approach. To verify whether chromosomes contribute in equal amounts to LTEEG and encoding genes, we determined the normalised contribution index, *i.e.* the relative contribution to LTEEG of a chromosome as compared to its relative contribution to protein-encoding genes (%LTEEG_chr_/%PEG_chr_). A contribution index of 1 indicates that the contribution to LTEEG is in accordance with the contribution to coding genes; a contribution > 1 indicates an enrichment.

### Determination of the relative abundance of X/Y-linked genes with the GO:0002376 annotation (GO approach)

The AmiGO 2 database [24] was browsed with the filters: Organism, *Homo sapiens;* Type, protein; and Biological process term, immune system process (GO:0002376) on October 25, 2019. The 3201 retrieved genes were downloaded and the chromosome distribution of the genes was obtained with use of e!Ensembl [19] or Genecards [20]. Eight genes obtained from the AmiGO 2 database were not found by the other gene databases and were excluded, so that the chromosome distribution of 3193 GO genes was determined. The AmiGO 2 database reported the gene segments of the chains of the B cell and T cell receptor as individual genes. We curated the gene number by considering gene segments of one chain as one gene. Thus, the gene number reduced to 2927 GO genes. The determination of the absolute and relative abundance of GO genes on the chromosomes and the normalised contribution index was analogous to the LTEEG work-up.

### Statistical analysis

Descriptive statistics was used to compare X/Y-linked ISRG, LTEEG and GO:0002376 genes. The Shapiro-Wilk test was used to verify whether the distribution of the immune genes over the chromosomes was normal.

## Results

### Gene populations

For the DEF approach, we analysed 882 protein-expressed genes (837 X-linked + 63 Y-linked - 18 PAR genes) to be annotated to system functions. For the LTEEG approach, the chromosome distribution was checked for 1391 LTEEG genes and 2927 genes for the GO:0002376 annotation (Fig. 1).

### Relative abundance of X/Y-linked ISRG as compared to genes dedicated to other system functions

**Fig. 2:**
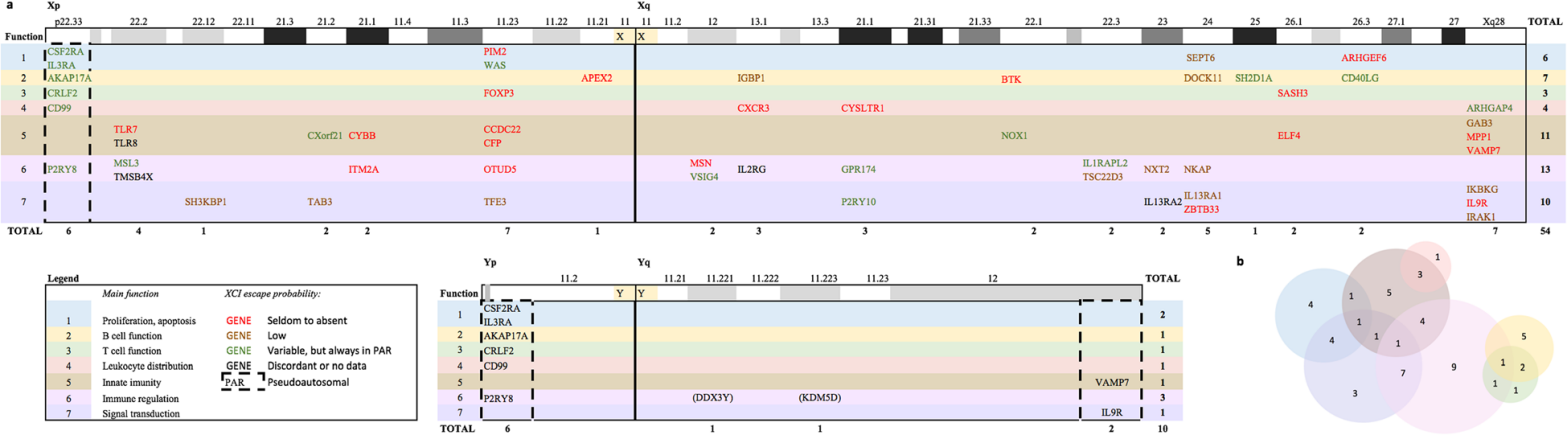
The distribution of immune system-related genes (ISRG) over the sex chromosomes. The genes are within colour-coded rows to indicate their main function. The font colour of each gene is according to their level of XCI or XCI escape probability and PARs are enclosed in a dashed box. Most ISRG involve various immune functions simultaneously, *e.g*. SASH3 may be a signal adapter in lymphocytes that regulates apoptosis and proliferation in both innate and adaptive immunity affecting both cellular and humoral immunity. Such cases were assigned to the main function in the distribution of ISRG over the sex chromosomes (**a**), but placed in the intersection of proliferation/apoptosis, innate and signal transduction in the Venn diagram (**b**). The Venn diagram should be considered the best possible approximation rather than an exact function annotation

A total of 56/882 (6.3%) X/Y-linked protein-expressing genes were annotated as ISRG, broken down as follows: 54/837 (6.4%) X-linked, 10/63 (15.9%) Y-linked, and 8/18 (44.4%) PAR-linked ISRG protein-expressing genes (Table 1, Fig. 2; Additional file 3). The 10 Y-linked ISRG were the 8 PAR-linked ISRG plus 2 candidate ISRGs, *i.e.* these genes did not comply convincingly with the annotation criteria, but we would like to mention them because of the impact they might have.

**Table 1:** System functions of X/Y-linked genes according to the DEF approach

Function	X	Y	nY/nX	PAR	nPAR/nX
	n (%)^r^	n (%)^r^	r	n (%)^r^	r
Basal/ubiquitous/unknown	335 (40.0)	11 (17.5)	0.03	3 (16.7)	0.01
Development, proliferation, apoptosis	87 (10.4)^3^	7 (11.1)^3^	0.08^3^	6 (33.3)^2^	0.07^2^
Immune system	54 (6.5)^4^	10 (15.9)^2^	0.19^2^	8 (44.4)^1^	0.15^1^
Nervous system	108 (12.9)^2^	2 (3.2)^4^	0.02^5^	0 (0)	
Endocrine system	35 (4.2)^5^	1 (1.6)^5^	0.03^4^	1 (5.6)^3^	0.03^3^
Reproductive system	153 (18.3)^1^	32 (50.8)^1^	0.21^1^	0 (0)	
Other systems	65 (7.8)	0 (0)		0 (0)	
TOTAL or average	837 (100)	63 (100)	0.09	18 (100)	0.06

* Other systems include the digestive, respiratory, cardiovascular, integumentary, urinary, and musculoskeletal systems; r, Rank. The classes ´Basal/ubiquitous/unknown´ and ´Other systems´ do not participate in the ranking to avoid bias due to undefined genes or because the genes may belong to different classes.

On the X chromosome, ISRG ranked fourth (Table 1). Genes dedicated to reproduction, the nervous system and growth/apoptosis/differentiation were more abundant. The endocrine system ranked below ISRG, and other systems were grouped together because genes dedicated to these systems were relatively scarce (Table 1, Additional file 1). On the Y chromosome, ISRG ranked second after genes dedicated to reproduction and followed by genes dedicated to development, proliferation and apoptosis. Only two Y-linked genes were dedicated to the nervous system and one to the endocrine system, whereas no genes were dedicated to other systems (with the exception of the ´Basal/ubiquitous/unknown´ category). The retention ratio on the Y chromosome, *i.e.* the number of Y-linked genes dedicated to a particular system as a fraction of X-linked genes dedicated to the system (nY/nX), ranked second for ISRG (Table 1), only after the reproduction-related genes, indicating a relative enrichment of ISRG on the Y chromosome. This was in stark contrast to genes dedicated to the nervous system, which are hardly retained on the Y chromosome. The enrichment of ISRG on the Y chromosome was mainly due to an enrichment of ISRG at the PAR. At the PAR, ISRG were the most abundant genes as compared to genes dedicated to other systems and ISRG also had the highest retention ratio (nPAR/nX) (Table 1).

Clearly, the distribution of ISRG over the sex chromosome was not homogeneous. Most Y-linked ISRG were at the PAR, with the exception of two candidate genes, *DDX3X* and *KDM5D*. X-linked ISRG concentrated (46.3%, 25/54) at p11.23, q24 and the chromosome extremes (Fig. 2). In summary, the sex chromosome-linked ISRG are not enriched at the X chromosome, but they are at the PAR, and therefore at the Y chromosome.

### Relative abundance of LTEEG and GO-immune system process genes on sex chromosomes

**Fig. 3:**
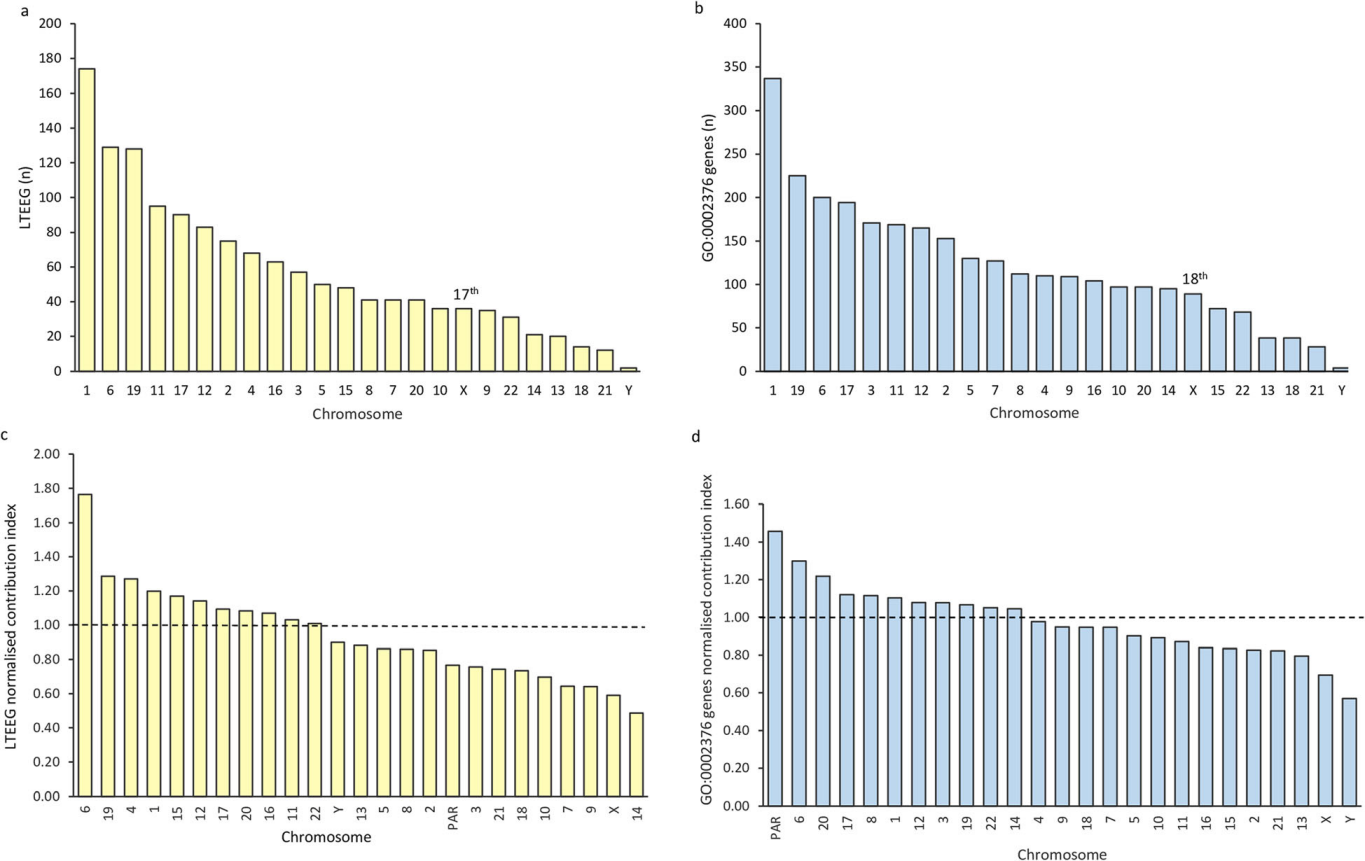
Immune genes and their distribution over autosomal and sex chromosomes. Immune genes were obtained because of either a 4-fold elevated expression in lymphoid tissues (LTEEG) or the annotation for immune system process (GO:0002376). Chromosomes are ordered by rank based on their absolute number of LTEEG (**a**) or GO genes (**b**). Likewise, chromosomes were ordered according to their normalised contribution index to LTEEG (**c**) and GO genes (**d**). The normalised contribution index of each chromosome is calculated as follows: %LTEEG_chr_/%PEG_chr_ , with %LTEEG_chr_ is the proportional contribution of the chromosome to all LTEEG and %PEG_chr_ is the proportional contribution of the chromosome to all protein-expressed genes (PEG). The dashed line at “1” indicates that a chromosome has the same relative contribution to LTEEG as to PEG

Both genome-wide approaches detected that chromosomes 1, 6 and 19 were enriched for immune genes (Fig. 3), whereas the X chromosome ranked 17^th^ according to the LTEEG approach (Fig. 3a) and 18^th^ according to the GO approach (Fig. 3 b). Even though the X chromosome contained more coding genes than the median of the autosomal chromosomes, the contribution of the X chromosome to LTEEG and GO-defined immune genes was less than the contribution from autosomal genes in every aspect: absolute number (Table 2, Additional files 4 and 5), relative contribution to immune genes (defined as LTEEG or GO_0002376 genes), proportion of immune genes among coding genes per chromosome, and the normalised contribution (Table 2 and Fig. 3 c and d, Additional files 4 and 5). The normalised contribution indices were 0.59 and 0.69 for the LTEEG approach and the GO approach, respectively (Table 2), which ranked the X chromosome at the penultimate position (Fig. 3 b and d). Thus, it seems that the X chromosome, rather than being enriched, has less immune genes than most autosomal chromosomes.

**Table 2:** Contribution of chromosomes or the PAR to genome-wide immune genes

	GW	Median autosomal	X	Y	PAR
Coding genes (n)	19186	777	841	46	18
Rel. contribution to coding genes (%)	100	4.05	4.38	0.24	0.09
LTEEG (n)	1391	49	36	3	1
Rel. contribution to LTEEG (%)	100	3.52	2.59	0.22	0.07
LTEEG_chr._/coding genes_chr_ (%)	7.25	6.85	4.28	6.52	5.56
Normalised contribution index	1.00	0.95	0.59	0.90	0.77
GO:0002376 genes* (GO) (n)	2927	111	89	4	4
Rel. contribution to GO (%)	100	3.76	3.04	0.14	0.14
GO_chr._/coding genes_chr._ (%)	15.25	15.34	10.58	8.70	22.22
Normalised contribution index	1.00	0.97	0.69	0.57	1.46

*GW* Genome-wide, *PAR* Pseudoautosomal region, *Rel.* relative, * curated number, i.e. gene segments of B and T cell receptor chains considered as one. Crude data and analysis (Additional files 3 and 4).

As expected, the Y chromosome and the PAR had the lowest absolute and relative numbers of immune genes (Fig. 3 a and b, Table 2). Unexpectedly, when corrected for the reduced number of coding genes on the Y chromosome and at the PAR, LTEEG and GO-defined immune genes scored higher than their X-linked counterparts (Table 2 and Fig. 3 c and d). When comparing between Y-linked and PAR-linked immune genes, the two genome-wide approaches differed. The Y chromosome scored higher with the LTEEG approach, while the PAR region scored better than the Y chromosome with the GO approach (Table 2, Fig. 3 c and d). The Y-linked immune genes detected by the GO approach were all PAR genes (Additional file 5), whereas the LTEEG approach detected two non-PAR Y-linked genes that were highly expressed in lymphoid tissue: *SRY* and *UTY* (Additional file 4). In summary, although the Y chromosome and PAR have the lowest absolute contribution to immune genes, they outperform the X chromosome when the reduced number of coding genes is considered.

### Approach comparison

**Fig. 4:**
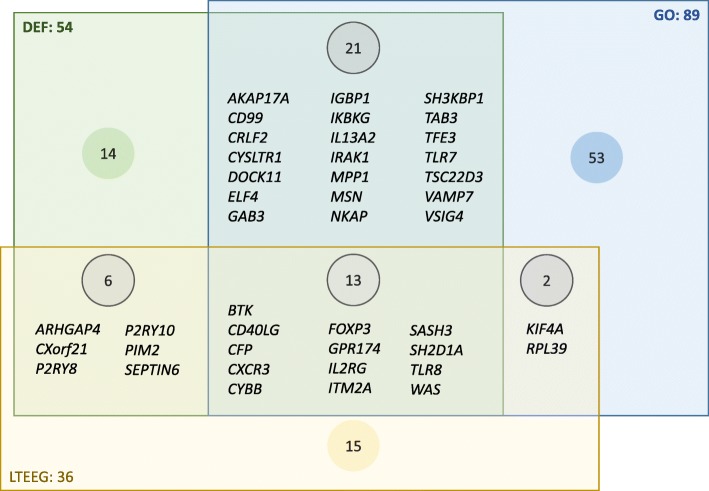
Diagram representation of immune genes detected by a single approach or varies approaches. The green box contains immune system-related genes (ISRG) detected by the DEF approach, the yellow box LTEEG, and the blue box GO genes. The number indicates the number of ISRG, LTEEG and GO genes detected by each method. Where overlap occurs, the confirmed immune genes are specified

Among the three approaches, 124 X-linked immune genes were identified, a third thereof were detected by at least 2 approaches (Table 3, Additional file 6). The number of X-linked immune genes detected by the three approaches varied greatly. The GO annotation approach detected the largest number (n = 89), but had the lowest proportion (40%) of confirmed immune genes. The LTEEG method had the lowest number (n = 36) and an intermediate proportion (58%) of confirmed immune genes. The DEF approach detected an intermediate number of immune genes (n = 54) of which 74% were confirmed. Thus, the DEF approach seemed to perform best. Among the 42 X-linked confirmed immune genes, 13 were detected by all three approaches, and 29 by two approaches (Fig. 4). All approaches discarded an enrichment of immune genes at the X chromosome (Tables 1 and 2, Fig. 3). Both the DEF approach and the GO approach detected that the PAR contained a relatively large number of immune genes among the few coding genes. Both the DEF approach and the LTEEG approach detected non-PAR Y-linked genes with a preferential expression in leukocytes, *KDM5D* and *UTY*, respectively (see also below, and Additional files 3 and 4).

**Table 3:** Approach comparison

	ISRG	LTEEG	GO	TOTAL
Immune genes detected by a single approach	14	15	53	82
Immune genes detected by >1 approach	40	21	36	42
TOTAL	54	36	89	124
Confirmed immune genes (%)	74	58	40	34

### Expression control of X-linked ISRG

All PAR1 ISRG for which expression data were available escaped XCI, but the expression pattern revealed a male bias. The expression levels of *CRLF2* were below the reliability threshold [29]. The two PAR2 ISRG were subject to XCI, but had different expression profiles; *VAMP7* expression was sex neutral, while *IL9R* had a male bias (Table 4). With respect to non-PAR X-linked ISRG, most (31/46, 67.4%) were subject to XCI, while 23.9% (11/46) variably escaped XCI and 8.6% (4/46) had discordant or unknown XCI escape data. Interestingly, independent of the XCI status, about two-thirds of the non-PAR X-linked ISRG were expressed without sex bias, about a quarter was expressed with female bias and a minority with male bias (Table 4). Thus, with respect to the 54 X-linked ISRG, 33 (61.1%) were expressed without sex bias, 11 (20.4%) with female bias, and 9 (16.7%) with male bias, (Table 4, Additional file 7).

**Table 4:** Abundance of sex-biased expression of X/Y-linked DEF-defined ISRG

		Sex bias, *n* (%)*			
Gene type and XCI status*	ISRG, n	No data	Male	Female	No bias
PAR	8	1 (12.5)	6 (75.0)	0 (0)	1 (12.5)
XCI escape (PAR1)	6	1 (16.7)	5 (83.3)		
XCI (PAR2)	2		1 (50.0)		1 (50.0)
Non-PAR X-linked *	46		3 (6.5)	11 (23.9)	32 (69.6)
XCI (mainly)	31		1 (3.2)	8 (25.8)	22 (71.0)^h^
XCI escape (variable)	11		1 (9.1)	3 (27.3)	7 (63.6)
Discordant /unknown	4		1 (25.0)	0 (0)	3 (75.0)
TOTAL X-linked	54	1 (1.9)*	9 (16.7)	11 (20.4)	33 (61.1)
Non-PAR Y-linked candidate ISRG	(2)	NA	2 (100)	NA	NA
Non-PAR X-linked paralogs XCI escape	(2)			2 (100)	

*XCI* X chromosome inactivation, *XCI* status and sex-biased expression according to Tukiainen [29]; *, there were insufficient data on the expression of this gene to properly analyse expression bias; ^h^, the numbers include *MPP1*, which displayed a heterogeneous sex bias

The non-PAR Y-linked candidate ISRG, *DDX3Y* and *KDM5D*, are obviously only expressed by males. Their non-PAR X-linked paralogue pairs, *DDX3X* and *KDM5C*, both escape XCI and are expressed with female bias. Importantly, the tissue expression pattern of the paralogue pairs differs. *KDM5D* is preferentially expressed in leukocytes, while its X-linked paralogue *KDM5C* is ubiquitously expressed [21]. Likewise, *DDX3Y* displays a markedly high expression among leukocytes, while the X-linked paralogue *DDX3X* is preferentially expressed in the male reproductive tract and leukocytes [21]. These Y-linked genes were considered *candidate* genes, because they did not comply convincingly with the annotation criteria. They only complied with the expression criteria from a single expression database: BioGPS [21]. Our argumentation to include them as candidate ISRG is provided in the discussion.

### Functional aspects of ISRG

With respect to the type of immunological functions encoded by ISRG, the largest group were immunoregulatory genes. With respect to innate and adaptive immunity, X/Y-linked were more often involved in the former (Fig. 2a and b). *FOXP3* was originally assigned to the immune tolerance, but being the single member, was reassigned to T-cell function. *CXorf21* was another doubtful case, but was annotated as ISRG with an immunoregulatory function with a special impact in the innate immune system because of two reports that identified *CXorf21* as an interferon-inducible gene involved in *TLR7* expression [32, 33]. Twenty-three possible X-linked genes raised doubts on whether to annotate them as ISRG, but were discarded because of insufficient consistency among expression data or insufficient support of a direct involvement in immune function or disorder (Additional file 2). For example, the association of *TAZ* with immune dysfunction is less strong than the one with cardiomyopathy. TAZ expression is preferential in the immune system according to BioGPS, but other expression databases indicate preferential expression in heart, muscle and endocrine tissues, so that it was assigned to the ´basket´ category: basal metabolism or unknown function [20]. Likewise, in other doubtful cases the main dysfunction was not related to the immune system, the main function was not limited to the immune system, and/or a minority of the consulted expression databases reported that a gene is preferentially expressed in lymphoid tissue, respectively indicated with D0.5, F0.5 and E0.5 in Additional file 2.

## Discussion

The absolute number of X/Y-linked immune genes varied greatly depending on the approach. In this study, the LTEEG approach, which was based on a four-fold elevated expression in lymphoid tissues as compared to non-lymphoid tissues [22], detected the smallest number. The simple, but strict criterion of the LTEEG approach has several weaknesses. First, the cut-off level of a four-fold expression level may be too strict for certain ISRG. Indeed, leukocytes are present in many non-lymphatic organs (intestinal and bronchial MALT lymphocytes, lung macrophages, brain microglia, hepatic Kupffer cells, etc.) so that a gene with a preferential expression in leukocytes may not stand out with this criterion. In contrast, the DEF approach screened for a preferential expression in lymphoid tissues or leukocytes without considering a cut-off level. Furthermore, many genes involved in immunity are only expressed upon a certain stimulus or activation process, which may be missed when screening is limited to normal/healthy, unstimulated tissues. In comparison, the DEF approach used a variety of datasets to verify expression levels which included BioGPS datasets on cells or tissues during different phases of development or stimuli [21]. In general, any high-throughput method that uses a reasonably, but still arbitrarily, chosen cut-off value runs the risk of missing cases or including irrelevant cases. Second, the LTEEG approach did not consider immune function nor associations with disorders of immunity, which may explain why it missed well-known immune genes detected by the other two approaches (*e.g*. *CD99, IKBKG, IL13RA2, IRAK1,* and *TLR7*).

After a first screening for ISRG, certain X/Y-linked genes were considered doubtful as they did not comply convincingly with at least one of the established criteria. When further investigation revealed that the only known function or disease was related with the immune system, and there were no arguments to be assigned to another system, the genes were annotated as ISRG, *e.g*. *CXorf21* [33], *DOCK11* [34], *GAB3* [35] (Additional file 3). All these were confirmed by the GO approach. On the other hand, *MOSPD2* remained a doubtful case because the DEF criteria suggested contradictory classifications (Disease: Development; Expression: did not pass Bgee filter; Function: ISRG but based on a single publication [36], *i.e.* not convincingly). Thus, when none of the DEF criteria was convincingly fulfilled, or criteria were contradictory the ´doubtful´ genes were discarded as ISRG (Additional file 2).

The GO approach detected the largest number of immune genes, reaching an amount similar to previous reports [15, 37], but it included basal metabolism/ubiquitous genes (*e.g.* X-linked *G6PD* and *VEGF*) (Additional file 5). Thus, each approach had its weakness; the GO approach for its low specificity, the LTEEG approach for its demanding cut-off criterion, and the DEF approach because of human interpretation. Those aspects were reflected in Table 3 where LTEEG detected the lowest overall number and GO the highest non-confirmed number.

However, rather than trying to establish the most correct number of X-linked immune genes, the aim of the study was to verify whether the X chromosome is enriched for immune genes. Hereto, the most important aspect of each approach was that it provided a comparative framework. As long as the weakness had an equal impact on X-linked immune genes and their context it would not affect the pattern. Thus, despite the weaknesses of each approach and the disparities in their numbers, the three approaches revealed the same pattern. According to our data, the X chromosome is not enriched for immune genes, which contradicts previous reports [15, 17, 30]. Unexpectedly, the Y chromosome conserved quite some immune genes, mainly at the PAR. The abundance of immune genes at the PAR is most evident in primates and humans, which have a shorter PAR1 than other mammals [38].

The question remains: is the *number* of immune genes on the X chromosome sufficient to explain the observed sex differences in immune responses? Several authors seem to think so [15, 17, 30, 39]. Rather than a simple yes or no, we believe that the control of gene expression is important. At the gene level, women (XX genotype) have twice the amount of X-linked genes as compared to men (XY genotype). Gene dosage compensation of X-linked genes is accomplished by XCI of one female X chromosome at random. However, as a vestige of the autosomal origin of the sex chromosomes and to facilitate the pairing of the X and Y chromosomes during male meiosis, X-linked genes have Y homologues at the distal ends of the sex chromosomes, at the PAR [38, 40]. To maintain gene dosage balance of PAR genes, X-linked PAR genes escape from XCI. However, gene expression from an inactivated X chromosome rarely reaches the same level as the one from the active gene [29], so that the expression of the PAR1 genes presents a male bias in most tissues [29, 40]. At least that is the general profile from an expression study across tissues, which included the spleen. Interestingly, the PAR2-linked *IL9R* and *VAMP7* do not escape XCI [28] and their expression seems differentially regulated as *VAMP7* is expressed without sex bias, while the expression of *IL9R* is male biased in many tissues [29].

With respect to non-PAR X-linked genes, most are effectively inactivated in most female tissues [28, 29]. But some of the non-PAR X-linked genes have an Y-linked paralogue. In such cases, XCI escape in the female is expected to ensure dosage equilibrium for the X-linked and Y-linked variants in the male [40]. In our study, both the *DDX3X-DDX3Y* pair and the *KDM5C-KDM5D* pair confirmed the predicted pattern. However, where *DDX3X* and *DDX3Y* have a similar expression distribution (mainly in gametes and leukocytes), the expression distribution of *KDM5C* and *KDM5D* differed. The X-linked version *KDM5C* is expressed ubiquitously, whereas the Y-linked *KDM5D* presents a preferential expression in gametes and leukocytes, which may either generate sexual dimorphism in leukocyte behaviour or be an attempt to neutralise gene dosage differences.

Among non-PAR X-linked, 20 ISRG presented a male or female sex bias, which may be important to explain the sex differences found in the human immune response. An interesting finding was that sex-biased expression among ISRG seemed independent from the XCI status. Indeed, among both XCI-subjected and XCI-escape ISRG, about two-thirds were expressed without sex bias and about a quarter with female bias. It should be emphasised that these expression data reflect a general expression profile across tissues, but the actual expression pattern in a specific tissue may be different, as has been reported for brain tissue [41] and for particular ISRG expressed in leukocytes. Indeed, the Epstein-Barr virus-transformed lymphocytes displayed a different expression bias of PAR1 genes than the other tissues [29]. And *TLR7*, an ISRG considered to be subject to XCI without sex-biased expression [29] has been reported to escape XCI and present female bias in a substantial fraction of biallelic immune cells as compared to male monoallelic leukocytes [16, 42]. Similarly, different from a male-biased expression pattern of *CD40LG* according to [29] (Additional file 3), stimulated leukocytes present a female-biased expression [16]. A similar situation has been reported for *CXCR3* [43] in activated T cells [44]. The aforementioned suggests that the general expression profile may not reflect the expression of a specific leukocyte or lymphoid tissue. Therefore, studies of sex-biased expression of X-linked ISRG should be performed in leukocytes or lymphoid tissue.

Still, male-biased expression of biallelic PAR genes has been reported for PAR-linked ISRG in the spleen [29]. This can be explained by incomplete XCI in women [29], but there may be other explanations. Male-biased expression of the non-PAR, single-gene ISRG, *CD40LG*, *TFE3*, and *TMSB4X* [29] seems counterintuitive. This phenomenon was independent of the XCI status. So that, even though an ISRG variably escapes XCI and can be expressed from both alleles in women, male expression bias has been reported, even in the spleen [29]. This could be explained by low intensity expression from either or both alleles in female cells, or, in males, single X-linked genes could be upregulated. This phenomenon has been described for *Drosophila* [45]. Upregulation of a single X-linked gene may also occur in mammals, including humans [41]. The latter could be achieved by a variety of mechanisms such as mRNA stability, translational and post-translational control mechanisms, and epigenetics. In this respect, the preferential expression in lymphoid tissue of a few non-PAR Y-linked epigenetic regulators is intriguing. Both *KDM5D* (detected by the DEF approach; Additional file 3) and *UTY* (detected by the LTEEG approach; Additional file 4) have histone demethylase activity, respectively for trimethylated lysine-4 on histone 3 (H3K4me3) and H3K27me3. *UTY* and H3K27 methylation seem to be involved in lymphocyte development in the thymus [46], while H3K4me3 is one of the epigenetic marks to escape XCI [45], as it is also the substrate of the *KDM5D* paralogue X-linked *KDM5C.* This paralogue pair displays a differential distribution profile as only the Y-linked version is preferentially expressed in leukocytes. Furthermore, *KDM5D* is known to regulate androgen receptor transcription by demethylation of H3K4me3, which is important not only in the reproductive system, but also for the function of androgen receptor-expressing leukocytes. The X-linked androgen receptor gene modulates the immune response [5]. *KDM5D* also interacts with the candidate Y-linked ISRG *DXD3Y*, X-linked ISRG *AKAP17A* [20]. Besides, in KDM5D-knocked down mice, *THEMIS2* was down regulated [47]. In humans, THEMIS2 is mainly expressed in leukocytes, especially B cells [48], and has a role in inflammation and the immune response [21]. Thus, Y-linked *KDM5D* may have an important role in controlling the expression of ISRG and either explain sex differences or neutralise them.

A variety of experiments could be performed to verify the importance of *DDX3Y*, *KDM5D,* and *UTY* for the immune response. These genes could be knocked-down in male leukocytes and its impact on the expression levels of X-linked ISRG determined. Or knock-out mice could be generated to verify expression of X-linked ISRG in lymphoid tissues and cells as well as the impact on the immune response in male mice. Besides, a variant of the four-core gene mouse model could be generated for these genes, similar to the one generated for the *SRY* gene [49]. This model would allow to compare the gene expression and functionality of normal female (XX) and male (XY) human leukocytes and recombined female XX_*KDMD5+/DDX3Y+/UTY+*_ and male XY _*KDMD5-/DDX3Y-/UTY-*_ leukocytes to determine whether Y-linked *KDM5D* and *DDX3Y* control the expression of ISRG. The four-core genotype mouse model is an elegant tool to study the effect of X/Y-linked genes, especially in gonadectomised mice. However, extrapolation of results from mouse models to humans is complicated as the regulation of XCI differs between mice and humans [50, 51].

Another epigenetic regulator that deserves attention is the X-linked *MSL3* gene product, which was preferentially expressed in leukocytes. In humans its function is unknown, but in Drosophila *MSL* genes are involved in equalising X-linked gene expression in males and females [52].

A recent, interesting finding is that dosage compensation occurred in certain mouse immune cells, despite the absence of Xist RNA (Xist is a long non-coding RNA that drives XCI) [51]. This finding supports the notion that apart from XCI, there are other mechanisms for gene dosage compensation.

Indeed, gene expression regulation of X-linked genes is complex and highly variable [53]. It involves epigenetics, partial inactivation, intraindividual mosaicism, age-dependent reactivation of previously inactivated genes [53, 54], cell-specific [51] and activation-dependent regulation [44]. The expression levels of X/Y-linked ISRG are further regulated by sex hormones, non-coding RNAs [31, 55], mRNA half-life [56] and a combination of these. These mechanisms may not only favour sexual dimorphism when needed, but also the opposite, *i.e*. ensure immune function with a minimum of sex difference.

In summary, the qualitative impact of X/Y-linked ISRG in the functioning of the immune system is difficult to predict. Expression regulation beyond Xist and XCI, including male mechanisms of expression control, should be studied in the cells of interest to elucidate the impact of X-linked genes.

### Study limitations

Although a strength of the DEF approach was the definition and impartial application of three criteria (disease, tissue expression and function) of which at least one had to be convincingly complied to be annotated to a system, doubtful cases could not be avoided. The most common reasons were: 1) the function of a particular gene is especially important for two different systems, 2) inconsistency in the annotation among the criteria (*e.g.* disease criterion is convincing for the nervous system, but the expression criterion for the immune system) or 3) none of the criteria was convincingly complied. The latter reason occurred most often, because genes had not been associated with a disease or their function was unknown. Furthermore, the information obtained from different databases on tissue-specific expression of a particular gene was not always consistent. Databases did not always report on the same tissues or organs. The ´human filter´ of the DEF approach was important to consider the special importance of the thymus, or the possibility that lung expression was actually localized in alveolar macrophages, and to verify whether a reported function was generally accepted or based on a single report. Additional file 1 does not reveal all considerations made for some genes, but Table 3 and Fig. 4 reveal that the DEF approach performed well. We feel that possible errors of judgment probably did not affect the final pattern of relative abundance, which seems to be a quite robust pattern.

A relatively large number of X-linked genes (40.1%) was ubiquitously expressed, had a system non-specific function, lacked an association with a system-specific disease or lacked data all together. Further knowledge on these genes may lead to a re-annotation that may affect the distribution of system functions of the X-linked genes. As such, the current annotation and relative frequencies is a function of current knowledge.

Rather than repeating the very laborious task of manual system annotation, which we performed for 882 X/Y-linked genes, for another 22,000 autosomal genes, we chose more feasible alternatives to contextualise the number of X-linked ISRG. Though we recognise that the internal reference method and the LTEEG and GO genes distribution are not the perfect controls to interpret the relative abundance of ISRG on the sex chromosomes, they both indicated that the X chromosome is not enriched for ISRG.

Our data do not support the viewpoint that the X chromosome is enriched for ISRG. Rather than the X-linked ISRG number, we recommend to consider other, probably more relevant, gene aspects, such as expression levels and the relatively large impact that a few particular X-linked genes may have on the immune response, *e.g*. *TLR7*, *TLR8* and *CD40LG* [16]. Furthermore, sex differences in the immune response may also be explained by sex-biased expression of autosomal genes. Indeed, a recent study to identify sexually differentially expressed genes in 11 immune cell types of C56BL/6J mice found that the majority of such genes were autosomal [57]. Still, the sex-biased expression of autosomal genes may be controlled, directly or indirectly, by genes on the sex chromosomes.

## Perspectives and Significance

Our comparative study revealed that the viewpoint that the human X chromosome has a larger number of ISRG than autosomal chromosomes is untenable. Unexpectedly, the Y chromosome and PAR contain a relatively large percentage of immune genes. Furthermore, Y-linked epigenetic regulators that have been involved in sexual dimorphism and immune regulation were preferentially expressed in lymphoid tissue. Therefore, we recommend to study the expression of sex chromosome-linked and autosomal immune genes in normal leukocytes and their subpopulations as well as the possible role of Y-linked epigenetic regulators in expression control.

## Conclusions

The supposed enrichment of ISRG on the X chromosomes was not supported by our data. Consequently, the viewpoint that the *number* of X-linked ISRG would influence immune responses is doubtful. The aforementioned does not deny that a sex difference in immune response could be due to particular X-linked immune genes, as seems to be the case for the X-linked RNA-sensors TLR 7 and 8. The PAR was remarkably enriched for immune genes, but as this region presents a gene dosage equilibrium between the sexes, the impact for sex differences in the immune response seems to be limited. The expression of X-linked genes seems highly regulated by mechanisms that go beyond female-specific XCI. For leukocytes, the role of *MSL3* deserve further investigation. And the preferential expression in leukocytes of the non-PAR Y-linked genes *KDM5D* and *DDX3Y* should be investigated for their role in the regulation of X-linked ISRG in the male. Regulation of X-linked ISRG, may be both to ensure sexual dimorphism of the immune system or, the opposite, to neutralise it. Expression studies of X/Y-linked ISRG should preferably be performed in a variety of human leukocytes and lymphatic organs to avoid extrapolation problems from data obtained in other tissues and animal models.

## Supplementary information

**Additional file 1.** XY genes, DEF data and manual system annotation. This Excel file contains 4 sheets: 1 for codes and numerical analysis, and 3 sheets for the DEF data collection on X-, Y-, and PAR-linked genes. It formed the basis for Table 1.

**Additional file 2.** Doubtful cases discarded as ISRG. This Excel file contains three sheets; the first for codes, the second provides 23 genes that were pre-selected as candidate ISRG, but did not pass the Bgee filter, and the third sheet presents a 79-item list of the 2008 Fish publication with updated information and the system code assigned by us, in accordance with Additional file 1.

**Additional file 3.** X-linked ISRG. This Excel file contains 2 sheets; the fist with a code key and the second with DEF data, XCI status and sex-biased expression for each ISRG. It formed the basis for Fig. 2 and Additional file 7.

**Additional file 4.** LTEEG data and analysis. This Excel file contains 3 sheets: the first with the gene-chromosome association, the second with the analysis, and the third presents excluded genes. It formed the basis for Table 2 and Fig. 3.

**Additional file 5.** GO0002376 data and analysis:.This Excel file contains 2 sheets: the first with the gene-chromosome association and the second with the analysis. It formed the basis for Table 2 and Fig. 3.

**Additional file 6.** Approach comparison. This Excel file contains 1 sheet that lists all X-linked immune genes detected by the three methods and shows per gene which method detected the gene. It formed the basis for Table 3 and Fig. 4.

**Additional file 7.** ISRG expression bias summary. This Excel file contains 1 sheet that corresponds with Table 4 within the main text and provides the specifications of Table 4.

## Data Availability

The datasets supporting the conclusions of this article are included within the article and its Additional files.
